# Smaller is better? Unduly nice accuracy assessments in roof detection using remote sensing data with machine learning and k-fold cross-validation

**DOI:** 10.1016/j.heliyon.2023.e14045

**Published:** 2023-02-24

**Authors:** Dávid Abriha, Prashant K. Srivastava, Szilárd Szabó

**Affiliations:** a Department of Physical Geography and Geoinformatics, Faculty of Science and Technology, Institute of Geosciences, University of Debrecen, Egyetem tér 1, Debrecen, 4032, Hungary; b Remote Sensing Laboratory, Institute of Environment and Sustainable Development, Banaras Hindu University, Varanasi, 221005, India

**Keywords:** Urban environment, Roof classification, Accuracy assessment, Salt-and-pepper effect, Post-classification, Object-based pixel purity

## Abstract

Deriving the thematic accuracy of models is a fundamental part of image classification analyses. K-fold cross-validation (KCV), as an accuracy assessment technique, can be biased because existing built-in algorithms of software solutions do not handle the high autocorrelation of remotely sensed images, leading to overestimation of accuracies. We aimed to quantify the magnitude of the overestimation of KCV-based accuracies and propose a method to overcome this problem with the example of rooftops using a WorldView-2 (WV2) satellite image, and two orthophotos. Random split to training/testing subsets, independent testing and different types of repeated KCV sampling strategies were used to generate input datasets for classification. Results revealed that applying the random splitting of reference data to training/testing subsets and KCV methods had significantly biased the accuracies by up to 17%; overall accuracies (OAs) can incorrectly reach >99%. We found that repeated KCV can provide similar results to independent testing when spatial sampling is applied with a sufficiently large distance threshold (in our case 10 m). Coarser resolution of WV2 ensured more reliable results (up to a 5–9% increase in OA) than orthophotos. Object-based pixel purity of buildings showed that when using a majority filter for at least of 50% of objects the final accuracy approached 100% with each sampling method. The final conclusion is that KCV-based modelling ensures better accuracy than single models (with better pixel purity on the object level), but the accuracy metrics without spatially filtered sampling are not reliable.

## 1 Introduction

The aim of image classification is to identify objects using remotely sensed data, and thematic accuracy is a fundamental requirement of classifications [[1], [2], [3]]. Several approaches can be chosen in order to accomplish the accuracy assessment (AA). (i) One of the earliest - and still an efficient - practice is simply to divide the reference dataset into a training and a testing subset. Usually 50–80% of the data is used for training the model and the rest for testing the outcome. Although this method provides valuable information about accuracy, partitioning is eventual as splitting reference data into training and testing groups is based on random selection. Consequently, another random split can result in a differently trained model and a different resulting map: if we conduct a classification with a given set of data, the final result has a high dependence on the reliability of the training dataset; i.e., non-representative pixels (representing the given class, but whose values become outliers in the set of collected reference data) can relevantly bias the classified map, and the accuracy indices (Overall Accuracy, User's Accuracy, Producer's Accuracy; Kappa Index of Agreement [4]) will reflect only the accuracy of this single solution. Supposing that training data can have inconsistency, and a single solution is just one possible outcome, the random selection can be repeated several times and, by running several models, we are able to judge whether a classified map is generated by chance or is reliable considering the statistical parameters. (ii) A possible solution is the increasingly popular *k*-fold cross validation (KCV) [5,6]. This technique splits the data into *k* equally sized subsets (usually 5 or 10) and, from these subsets *k-1* parts are used for training the models and the remaining ones for testing (supposing ten equal subsets the ratio is 9:1). The procedure is repeated *k*-times and finishes when each unique subset has been used for testing once. Overall Accuracy (OA) of the models is calculated *k* times from which various statistical metrics (mean, median, standard deviation, confidence interval or quartiles) can be calculated. (iii) A further improvement of this approach is the repetition of the KCV: the whole procedure is repeated with 3–5 further random samples (i.e., whole new datasets are generated from the original reference data). This approach is called the repeated *k*-fold CV method (RKCV [7,8]). Finally, statistical parameters such as mean or median OA and the deviation are based on 30–50 models. If the deviation (e.g., confidence interval) is narrow, the classification is reliable, but when it is wide, the outcomes are very dependent on the chosen set of training data; consequently, the training dataset cannot be considered reliable [9].

The most important point in AA is the reference dataset itself: beside the general requirements (e.g., representativeness, sufficient amount of data etc.), training and testing subsets should be independent. However, fine spatial resolution causes spatial autocorrelation (SA) [10]: pixels in adjacent position will be similar, e.g., a roof of a house can be in one pixel with 10 m or in 10000 pixels with 0.1 m resolution, thus, metrics of AA can be biased if SA is present. The concept of SA reflects Tobler's law of geography: similarity between objects increases as the distance decreases [11]; i.e., the objects of the dataset are not independent of each other, neighboring data have an influence on other data which is close or juxtaposed. SA is especially common for remotely sensed images where adjacent pixels usually relate to each other. Evaluating the model when the test dataset is not independent of the training dataset leads to the overestimation of the classification accuracies, due to the lack of randomness in consecutive or contiguous sets of data, i.e., the value of one data depends on the value of another. Therefore, reference data needs special attention to prevent the production of falsely good results. This phenomenon is a direct consequence of the RKCV-based AA of remotely sensed data with built-in algorithms in software: reference pixels are randomly split into training and testing sets; thus, the independency of the data used for training and testing cannot be ensured.

Remotely sensed data is highly biased by the SA [12]: when reference data is delineated as polygons on the pixel groups of satellite images or aerial photographs, random selection can choose pixels from the same polygon, both for training and testing. Moreover, adjacent pixels can belong to the training and the testing group, which violates the assumption of independence [13,14]. Theoretically, SA is not a problem when we collect object-based data, i.e., pixel values are aggregated by segments of similar pixel values as means, medians etc.; thus, training and testing data cannot be made up of adjacent pixels; accordingly, they should be independent. In this latter case, the results of the KCV-based AA are reliable. While the independence of training and testing data can be guaranteed easily in the case of simple AA by collecting a separate training and testing dataset, or using points instead of polygons, repeated k-fold CV as a modern AA-technique is biased. Given that in this case there is no separate training and testing dataset, the procedure is based on random data selection from the whole set of reference data, so the independence should be ensured in a special way.

Although RKCV is well-known in data science, it is still a relatively new technique in remote sensing, and implementation in remote sensing software is limited (k-fold cross-validation is implemented only in EnMap-Box 3, but without the possibility of repetitions). Thus, RKCV is only now becoming widespread in remote sensing [13,14]. R and Python packages can be efficiently used for this purpose, with several advantages: they are open source, flexible and models can be applied on any independent data regardless of the images and the areas; however, the main limitations of SA should be considered. Several authors examined the applicability of RKCV on highly autocorrelated data from the perspectives of hyperparameter tuning, spatio-temporal prediction, soil damage and water permeability prediction, and concluded that autocorrelation biases the accuracy metrics; therefore, training and test data should be spatially separated to avoid overestimation of thematic accuracy [[15], [16], [17], [18], [19]]. However, due to the calculation procedure of RKCV, completely independent testing is not possible; thus, in order to use it in remote sensing, as an informative method of AA, SA has to be eliminated directly in the reference data. Wadoux et al. [20] applied the design-based estimation approach, which was found to be better than spatial CV strategies, but this method cannot be used for clustered objects (e.g. rooftops, buildings, distributed habitat patches, etc.). Segmentation and point-based sampling can reduce the effects of SA, but there are cases in which the number of objects to be identified has a limited spatial extent, and these methods would relevantly reduce the possible number of reference pixels; thus, spatial independence should be considered differently. Despite the increasing number of applications of RKCV in remote sensing, there is no comprehensive quantification of the effect of SA, and the consequences are not evaluated.

Our target objects were roofs which are different from the regular land use/land cover mapping (i.e., mapping the whole studied area including all possible land cover units, and collection of reference data is not limited to given objects with a limited area): small surfaces with diverse appearance due to roofing materials, different aspects of roof planes (effect of sunshine), ageing of the materials, moss and lichens, and roof windows and chimneys while non-roof areas are masked from the image. The aim of this study was to quantify the level of bias on RKCV-based classification accuracy due to spatial autocorrelation, and to develop a method in Python which assorts the data based on the spatial location: data points inside a specified threshold are omitted from the analysis. We had the following assumptions: (i) effect of SA is the highest with the traditional and RKCV AA techniques related to AA-metrics of a completely independent dataset, (ii) finer resolution results in higher SA leading to falsely high accuracies, (iii) a spatial-based sampling approach helps to avoid the influence of SA, (iv) in spite of too optimistic accuracy measures, RKCV still ensures good accuracy on object level.

## 2 Methods

### 2.1 Study area

**Fig. 1:**
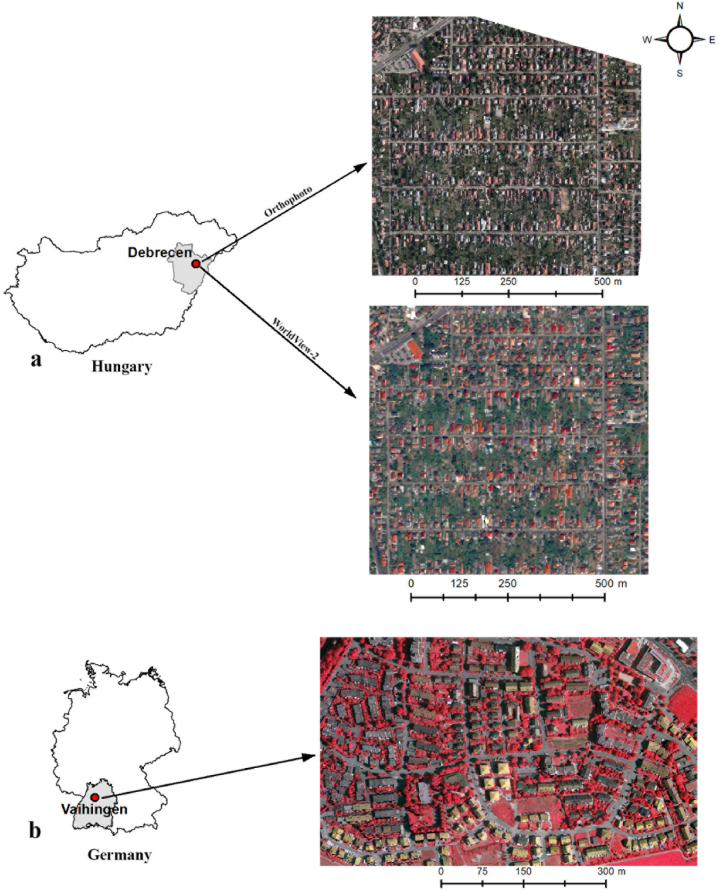
Location of the study areas in Hungary (a) and Germany (b).

Analyses were conducted in two areas: (1) Debrecen, the second largest city in Hungary (Fig. 1a) [21] and (2) Vaihingen an der Enz, a smaller town near Stuttgart, Germany (Fig. 1b). The Hungarian study area was dominated by detached houses, while in Vaihingen there were both detached and terraced houses. Brown and red tiles are the most common roofing materials in both areas, but in Debrecen asbestos roofs also occur in large quantities. Asbestos poses a high health risk as it is a carcinogenic material and causes cancer and diseases in the respiratory system [21]. Local authorities usually do not have a register of these asbestos roofs; thus, the environmental issues are not monitored [22]. In Vaihingen flat roofing is the main roofing type for terraced houses.

### 2.2 Datasets

We conducted the analyses on three different datasets and different study areas: two areas from Debrecen and one area from Vaihingen. In Debrecen, we used a WorldView-2 (WV2) satellite image and an aerial orthophoto to perform the investigations.

The WV2 image was captured on July 24, 2016 and had 8 spectral bands (coastal, blue, green, yellow, red, red-edge and two near-infrared) with a geometric resolution of 2 m. A panchromatic band was also available with 0.5 m resolution. We applied pan-sharpening on the multispectral bands to improve the geometric resolution to 0.5 m with the Gram-Schmidt pan-sharpening method [22,23].

The orthophoto was acquired with a Leica RCD 30 RGBN 60 MP camera in August 12, 2013 by Envirosense Ltd. The image had 4 spectral bands (blue, green, red and near-infrared) and a geometric resolution of 15 cm.

The image from Vaihingen (Germany) was a publicly available orthophoto from the WGII/4 2D Semantic segmentation benchmark dataset provided by the International Society for Photogrammetry and Remote Sensing (ISPRS; [24]). Geometric resolution is 9 cm and 3 bands are available: green, red and near-infrared. The image was captured in August 2008 with an Intergraph's Z/I Imaging Digital Mapping Camera system.

Reference data was collected during field surveys with a Stonex S9 RTK GNSS device in Debrecen. We identified the roofing types of 320 houses (all houses in the district except those having different roofing materials than the studied one, due to low instances) in the study areas using field observations and Google Street View. As there were four- and seven-year differences in time–for the WV2 and the orthophoto between the capturing of the images and the field survey–a few of the collected data had to be omitted due to the change in the roofing materials. Three types of roofing materials were collected: red tiles, dark tiles and asbestos cement panels (although we found other materials, such as metal or tar, these types were omitted from the analysis due to their low level of occurrence). We used the satellite imagery of Google Earth and Google Street View to identify the roofing materials in Vaihingen. Unlike in Debrecen, asbestos roofing was not present in this area, but we detected a large number of flat roofs, making it the third category after red and dark tile. Data were obtained from 50 houses for each roof type (almost all houses in a section where roofs were similar to the studied district of Debrecen). The collected data were digitized as polygons in the ENVI 5.3 software. Final reference datasets were obtained by pixel sampling with different approaches (detailed in section 2.4) within the rooftop polygons.

We created a building-mask layer for all three images. First, we derived Digital Surface Models (DSM) and Digital Terrain Models (DTM) from corresponding LiDAR surveys in each study area. We subtracted DTMs from the DSMs and produced the Normalized Digital Surface Models (NDSM) representing the relative height of the objects [24]. In addition, we constructed Normalized Difference Vegetation Indices (NDVI) from the red and NIR bands of the image sets [25,26]. The final mask is designed to include objects above 3 m and below 0.1 NDVI.

**Fig. 2:**
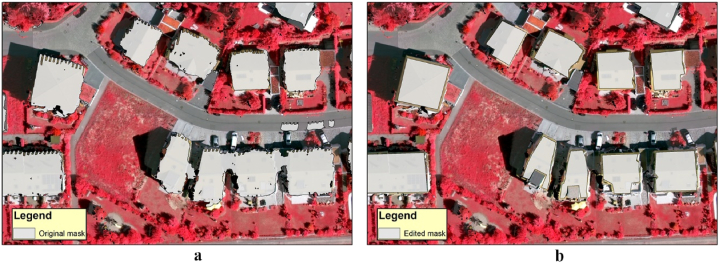
Result of the building masking (a: original mask; b: edited mask; background image: orthophoto of Vaihingen).

Although these masks provided mostly accurate results, several factors, such as the different imaging dates (e.g., the LIDAR survey was taken in 2013 and the satellite image of Debrecen in 2016), required manual editing of the masks to obtain better results (Fig. 2a,b). Once the masks were finalized, each building was assigned the appropriate roofing material to be used as a reference for further analyses.

Input datasets had different spectral resolutions, but following our aims, we focused on the differences in classification accuracy of the models, which were independent of the bands of the images.

### 2.3 Classification

Many studies showed that the Random Forest (RF) is a quite effective algorithm for image classification with the advantages of their non-parametric nature and high classification accuracy and capable of deducing variable importance [[25], [26], [27]]. Mishra et al. [28] compared various classification algorithms, such as maximum likelihood, neural network, RF and support vector machines in order to identify the best one for land cover classification, and concluded that the support vector machines and RF give better performance as compared to neural networks and maximum likelihood.

Due to the higher performance of RF, we also used here the RF algorithm for the classification [[29], [30], [31], [32]]. RF is a widely used and efficient method which belongs to the group of ensemble learners. The basic idea behind ensemble learning is that a set of classifiers provides better and more reliable results than individual ones [27]. The RF model is parameterized with the *ntree* (number of trees) and *mtry* (the number of variables selected at each split of the tree). In our case we had 8 predictors (i.e., bands of the satellite image) so in order to select an adequate value for the parameter, first we had to optimize it (hyperparameter tuning) [33,34]. The optimization was performed with the grid-search method in the Python programming environment and the *mtry = 4* was selected. RF incorporates hundreds of separate decision trees (*ntree*), usually between 300 and 1500, where each tree represents a prediction: the class with the most “votes” wins. Although it has less importance for the model's performance than *mtry*, selecting an inadequate number of trees can cause problems as it can lead to overfitting. We built the models with 300 trees. Image classifications were performed in Python 3.7 with the scikit-learn package [35].

### 2.4 Validation methods

Quantification of the model performance was the basis of the analysis; accordingly, we determined the overall accuracies (OAs) as a widely used measure of accuracy assessment [4,36,37]. OA is calculated by dividing the sum of correctly classified pixels by the total number of reference pixels. We used two terms for accuracies: (i) estimated accuracy calculated from cross-validation, and (ii) true accuracy obtained by validating with independent test data.

(i) Repeated *k-fold cross validation (RKCV)* is considered a robust estimator as it evaluates the performance of the classifier based on numerous models (repeats × folds). However, when class imbalance is present in the dataset, a variation of RKCV, and the *stratified k-fold cross validation (SCV)* can be used in order to minimize the possible negative effect of the uneven distribution on the results: SCV ensures the same proportion of features for all classes, i.e., class distribution of the selected data is equal among the folds. We applied 5-fold SCV with 10 repetitions to validate our results. Thus, all accuracy measures were derived from 50 models, and we calculated the median and quartiles of the models.
(ii) One of the earliest validation techniques is when model performance is evaluated with an independent part of the reference dataset. Accordingly, we collected data directly for an independent test set without any overlap or adjacency to the training data to ensure the independency (IT).

### 2.5 Experiments with different sampling approaches

First, we divided the reference dataset into a training and a testing part as a common way of model building and validation [6,38]. In this case, the model training and evaluation is based only as a single event (i.e., a single split). We split the dataset 80–20% for training and testing (the ratio was chosen to ensure similar conditions to the 5-fold cross validation), respectively; however, in order to make this method statistically comparable with SCV, where the outcomes were derived from 50 models, we repeated the procedure 50 times, randomly selecting a new training-testing subset for each repetition (TT).

**Fig. 3:**
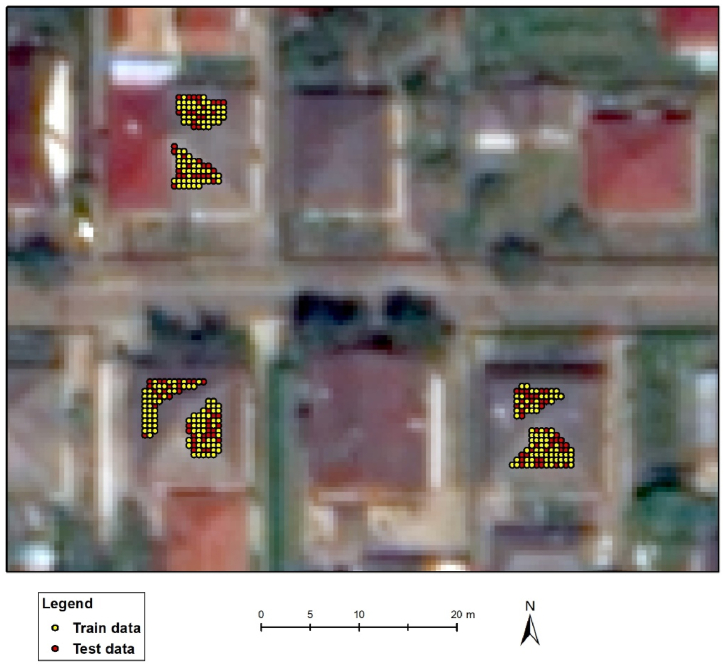
Visualization of training and testing data based on random selection at a ratio of 80–20 as possible folds of a 5-fold cross-validation method (background image: WorldView-2).

Cross-validation methods provide a good estimation of the model's actual performance if the dataset is consisted of independent data. However, satellite imagery is heavily affected by SA, because adjacent pixel values are similar [39]. This a common feature of remotely sensed images and can call into question the results of cross-validation: CV-based accuracy measures reflect falsely good outcomes if the reference data is not selected properly. Accordingly, in our case, a simple random selection from the reference data is not acceptable because points selected for training and testing are adjacent pixels having similar values; thus, the testing dataset will not be independent of the training data (Fig. 3). The higher the SA, the higher the bias on the accuracy measures.

**Fig. 4:**
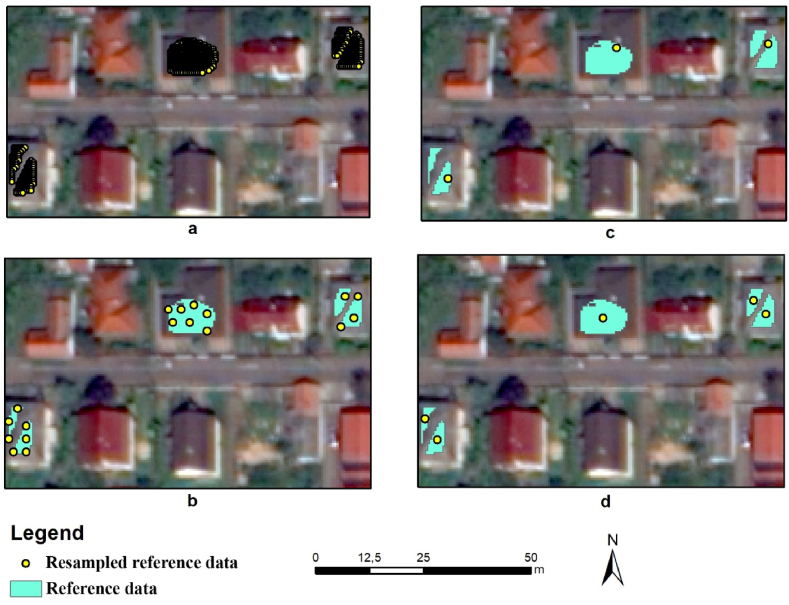
Spatial sampling based nt he method developed in Python with different distance thresholds (a: no threshold, CV; b: 2 m, SCV2; c: 10 m, SCV10; d: centroid, CVCent; background image: WorldView-2).

A simple 5-fold CV validation technique repeated 10 times applied on roof classification faces the same problem as presented above because it randomly splits the data into five folds without regard to spatial aspects (CV; Fig. 4a). Therefore, in order to solve the issue of CV and take into account the spatial features, i.e., to provide a spatial cross-validation approach suitable for estimating the actual accuracies of the models, we developed a new method in Python to eliminate the effects of SA. This approach assorts the reference data based on its spatial location: only points outside a specified distance (in meters) are selected. We used three different distance parameters: 0 (CV; Figs. 4a), 2 (SCV2; Figs. 4b) and 10 m (SCV10; Fig. 4c). With the increasing of the minimum distance between data points (Fig. 4b and c), the number of selected pixels decreased, and there were cases when no data was selected from certain polygons; therefore, we also analyzed the case in which all polygons were involved with one data point (using the centroids); classifications were run with 1 pixel/polygon (CVCent, Fig. 4d).

Furthermore, we also tested the Object Based Image Analysis (OBIA) approach. Segmentation was conducted with a seeded growing algorithm, first introduced by Adams and Bischof [40], which is a robust, rapid segmentation technique that does not require parameter-tuning. The neighborhood parameter was set to the Neumann approach, which uses 4 neighbors around a central cell in the four orthogonal directions. Lemenokva [41] found that in urban areas, the Neumann approach yielded good results; thus we also chose this method in our study using it with SAGA GIS 7.9 [42].

After the segmentation of the original image sets, the segments were clipped by the polygons of the reference data. It was an oversegmented layer, delineating spectrally homogenous areas, which did not reflect the entire objects (i.e. buildings) due to inhomogeneities such as shadowed roof planes and roof windows. Finally, one point was selected from each segment to run the classifications and one point per segments were involved in the classification with the RKCV (5-fold with 10 repetitions), too (CVSeg).

**Fig. 5:**
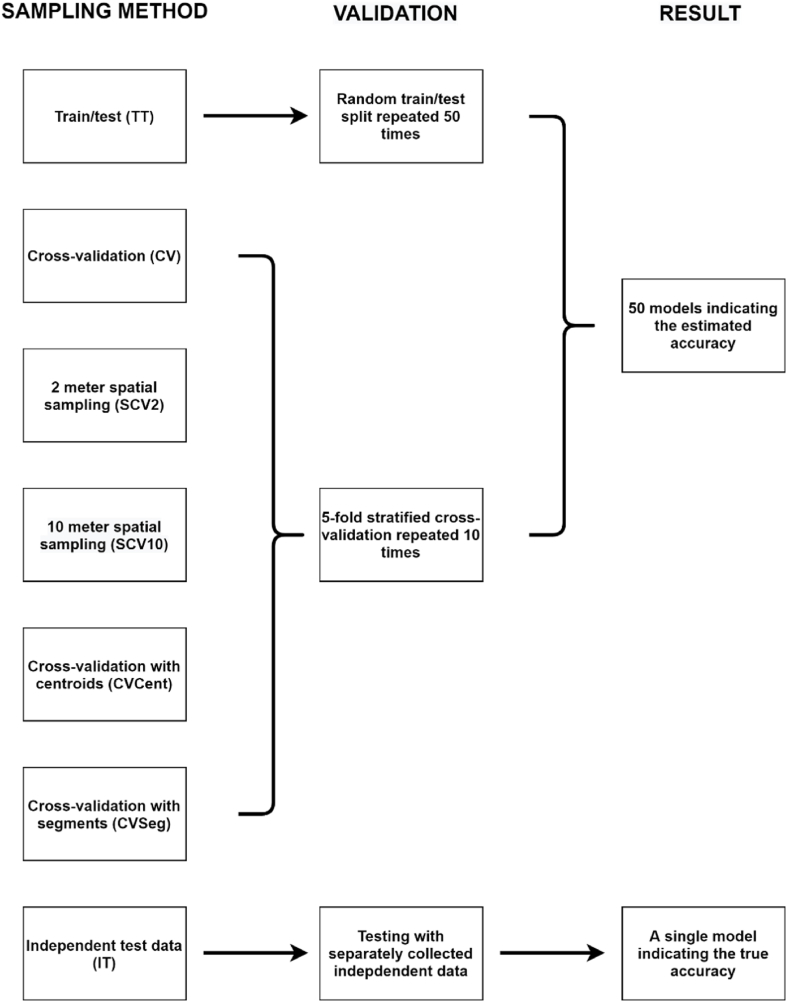
Workflow of the model evaluations by the type of sampling and validation methods.

Altogether, 7 types of sampling method were tested, and the validation was split randomly between training and testing data with 50 repetitions and 5-fold cross-validation with 10 repetitions, except for independent testing (IT) with a completely independent testing dataset representing the control, and all results were compared to this value (per experiment) (Fig. 5). We also determined the number of training pixels, because the selection also directly reduced the size of the data involved (Table 1).

**Table 1:** Number of evaluation pixels by input images.

Type	WV2	Ortho	ISPRS
IT	6200	15805	55216
TT	4340	11064	38651
CVSeg	357	1970	3301
CVCent	98	88	90
SCV2	268	253	122
SCV10	47	83	38

### 2.6 Evaluation of the effect of spatial autocorrelation

Moran's I (MI) was used to quantify the level of SA [43]. MI is one of the most prominent statistical metrics to express SA in a quantified form and is widely used in various GIS related research studies [[44], [45], [46], [47]]. The value of MI is between −1 and +1, where negative values indicate dispersion while positive ones suggest clustering tendencies [48]. We calculated the Mis in ArcGIS 10.8. Weights were row standardized, meaning each weight was divided by its row sum in order to obtain values between −1.0 and 1.0 [49]. Standardizing the weights was necessary as the distribution of the features are biased due to the nature of the data. Values were divided into 3 categories: -1 – 0.4 indicates low, 0.4–0.7 medium, and 0.7–1 high positive SA [50]. As our intent was to examine the positive SA and in remote sensing data adjacent pixels are usually highly correlated, we merged low positive SA values with the negative values.

We applied Generalized Linear Modelling (GLM) as a covariance model. OA was considered the dependent variable while the sampling technique and MI were involved as independent variables. Effect sizes (ω^2^p) were also calculated to quantify the importance of the independent variables in the equation in a standardized form [51]. The normality of residuals were checked with the Shapiro-Wilk test.

### 2.7 Object-based pixel purity (OPP)

**Fig. 6:**
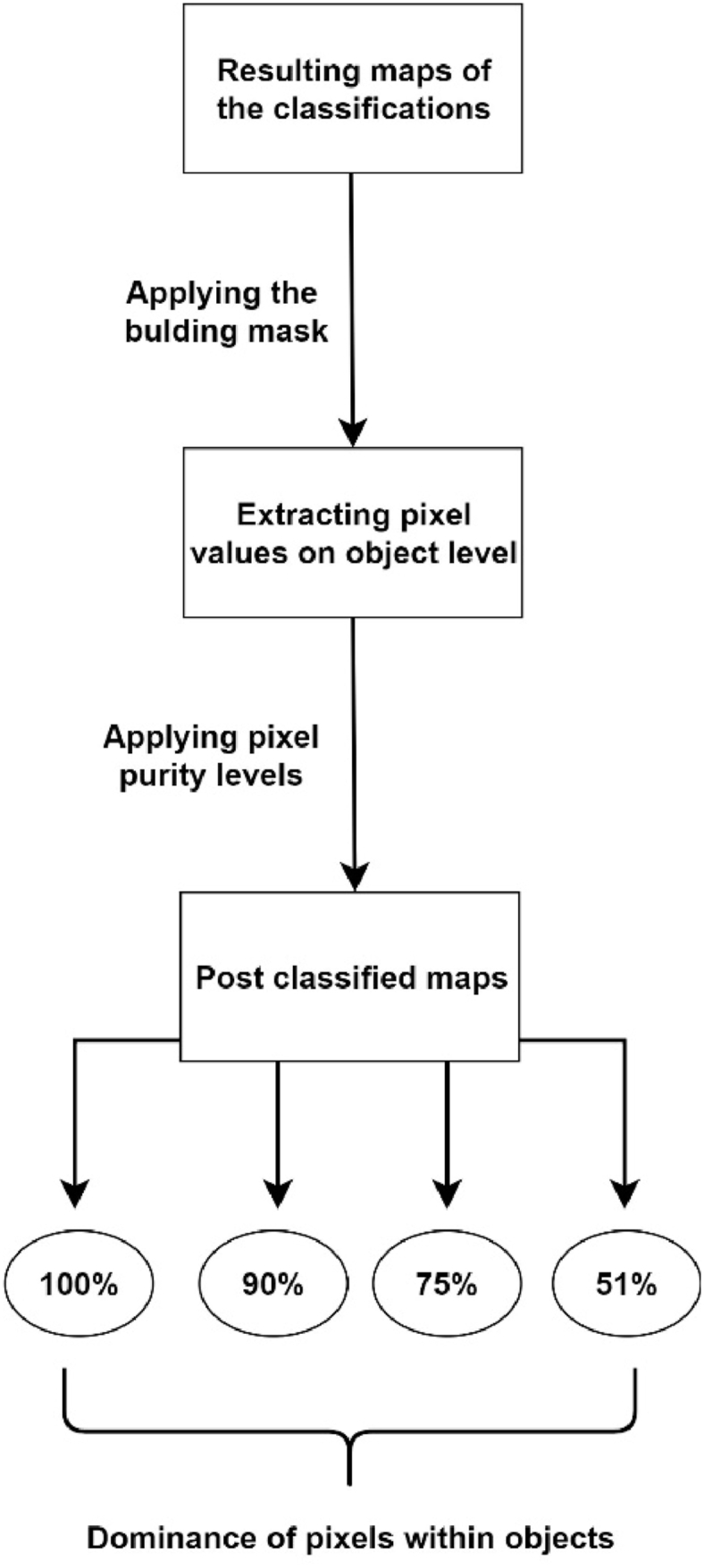
Steps taken to calculate object-based pixel purity.

Training and testing based on pixels provide an efficient way of justifying the outcomes, although they are limited to the pixels of the reference dataset. However, we also intended to reveal the accuracy on the object level; i.e. how many pixels were correctly classified within the buildings (Fig. 6). First, we defined the building mask (described in section 2.5) and extracted the pixels within the building polygons. We then evaluated all outcomes of different classification approaches on the object level, summarizing the ratio of correctly classified pixels (real roofing types were confirmed by field observation) related to all pixels of a given object (i.e., building). We defined four object-based pixel purity (OPP) classes at 100, 90, 75 and 51%. A purity of 100% means that the algorithm classified all the pixels of a given roof into the correct category, while 51% indicates that the majority of the pixels belong to the correct class. If at least 51% pixels were classified into the class in which the given building actually belonged, it could be considered a correct hit. These calculations were performed only on the datasets of Debrecen because classes were different (i.e. the asbestos was not present) in the case of Vaihingen.

A GLM was applied as a 2-way factorial ANOVA model to reveal the effects of the validation technique and the roofing type, and their statistical interaction on the OPP values as the dependent variable.

## 3 Results

### 3.1 Accuracies achieved by different sampling methods

**Fig. 7:**
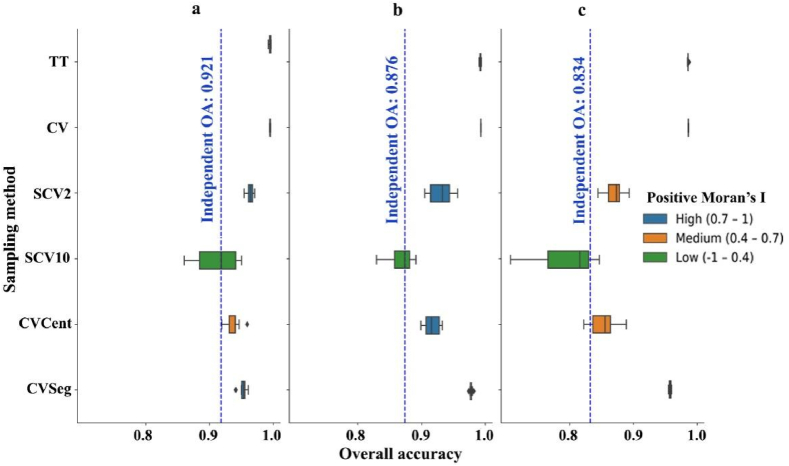
Classification results of the sampling methods based on 50 models with regard to Moran's I (a: WorldView-2; b: orthophoto of Debrecen; c: orthophoto of Vaihingen).

TT and CV both yielded similar outcomes for all three input datasets: mean Oas were above 98.5% with a narrow interquartile range (Fig. 7). Oas based on the CVSeg method were also overestimated (95–97%) related to the independent testing. Involving the distance parameters (SCV2 and SCV10) caused the decrease of the Oas to the IT level, but in this case the lower amount of data trained the models with the less SA, i.e. the reliability increased, but the interquartile ranges increased, too. Classifications using the centroids resulted in accuracies between the SCV2 and SCV10 models.

### 3.2 Spatial autocorrelation and sampling techniques

**Fig. 8:**
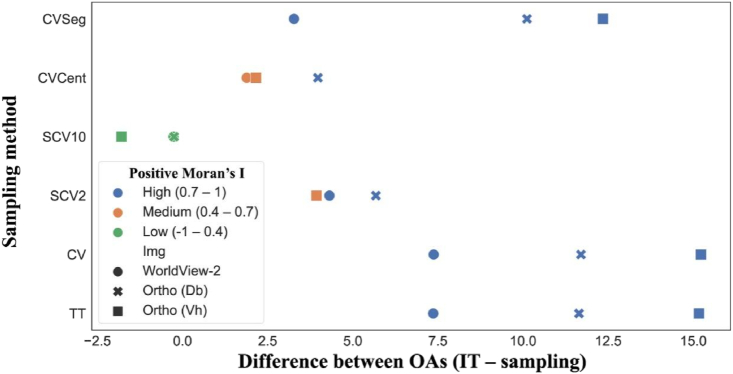
The differences between the mean overall accuracies of the models and the results of the independent testing.

Models had varying Mis in the function of input data, sampling technique and Oas (Fig. 8). The highest Mis were experienced with the highest Oas, and the correlation between MI and OA was 0.821 (p < 0.001). TT, CV and CVSeg followed the same pattern with the highest Mis; with the lowest (but still highly spatially correlated) values for WV2. The lowest Mis were only achieved with SCV10; furthermore, for each image this was the sampling method that efficiently minimized the differences between the estimated and the true accuracies. SCV2 and CVCent samplings were able to decrease the SA only to a medium level, whereas CVCent was more efficient.

GLM revealed that the sampling technique and the MI explained 73.2% of the variance of the OA (Table 2). All variables were significant (p < 0.001) and had large effect based on the ω^2^p. The sampling technique had the largest effect (0.712), and although the MI's effect was also large, it only accounted for one-third of the sampling technique.

**Table 2:** Result of GLM performed on overall accuracies as the dependent variable, and the input data type and sampling technique as independent variables (SS: sum of squares, df: degree of freedom, F: F-statistic, p: significance, ω^2^p: effect size).

Parameters	SS	df	F	P	ω^2^p
Model	0.0889	6	82.94	<.001	0.732
Sampling technique	0.0439	5	9.22	<.001	0.712
Moran's I	0.0450	1	47.25	<.001	0.204
Residuals	0.1647	173			
Total	158.4661	180			

### 3.3 Assessing the purity of the classifications based on the building masks

**Fig. 9:**
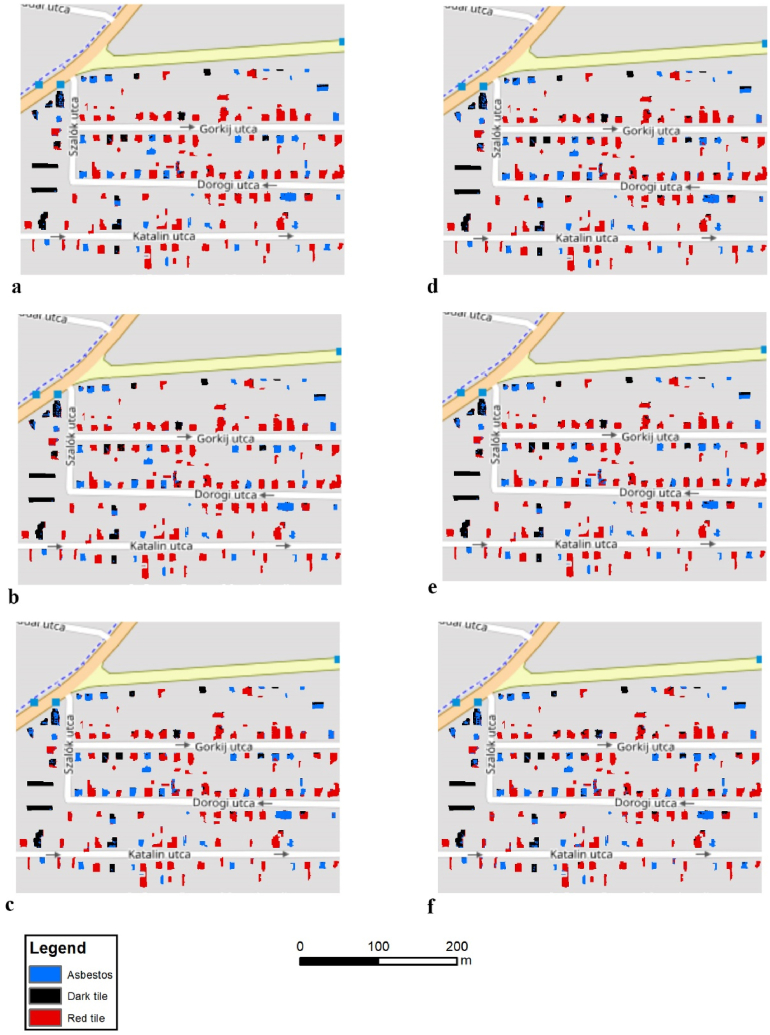
Classification results of roofing materials using the WorldView-2 satellite image in Debrecen by sampling methods (a: IT; b: TT; c: CVSeg; d: CVCent, e: SCV2, f: SCV10).

**Fig. 10:**
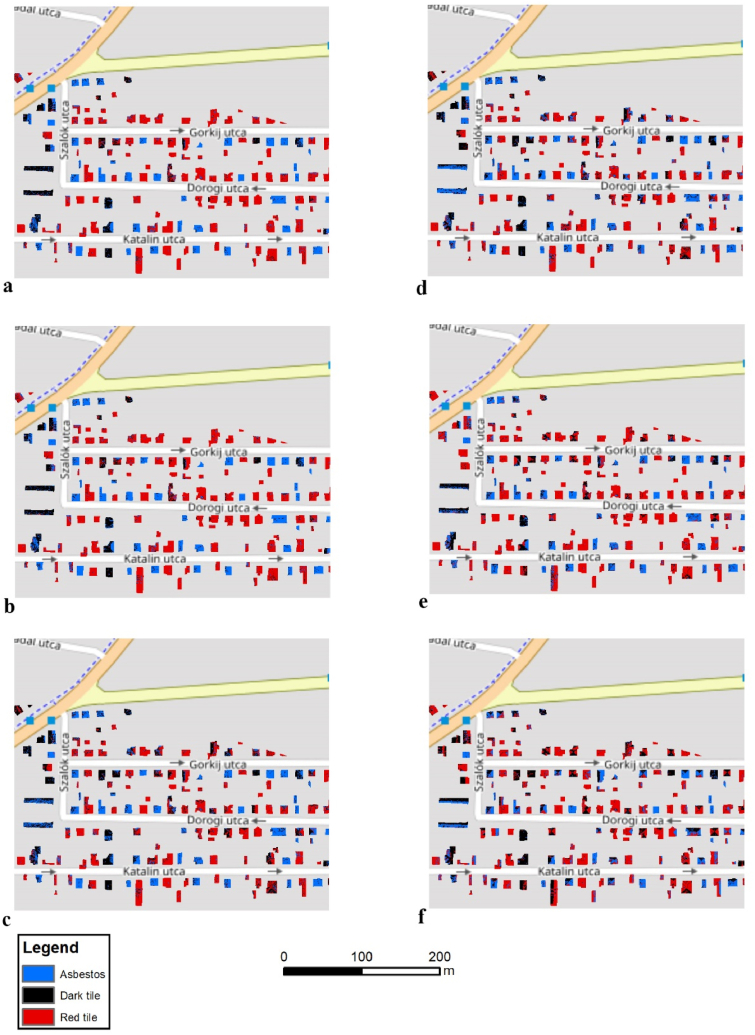
Classification results of roofing materials using the orthophoto in Debrecen by sampling methods (a: IT; b: TT; c: CVSeg; d: CVCent, e: SCV2, f: SCV10).

Pixel purity varied by the applied validation techniques, the input data, and the roofing types (Fig. 9, Fig. 10f). According to visual evaluation, the best solution, both in the case of WV-2 and orthophoto, belonged to the IT model with mostly purely classified buildings, but the other models also showed acceptable results. Some buildings had relevant interspersion; the classified pixels interspersed and usually the dark tile and the asbestos mixed within these objects, but in a few cases pixels of all the three types occurred. Usually, the level of misclassification was below 50% and the final class was correct after the application of an object-based majority filter.

**Fig. 11:**
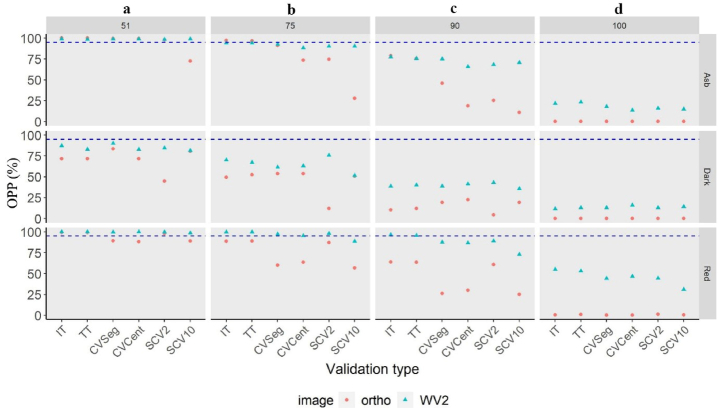
OPP (%) in the function of purity classes (51-75-90-100%; a-d), validation types (IT: independent testing; TT: train/test; CVSeg: cross-validation with segments; CVCent: cross-validation with centroids; SCV2: cross-validation with 2 m spatial sampling; SCV10: cross-validation with 10 m spatial sampling), roofing materials (Asb: asbestos, Red: red tile, Dark: dark tiles), and input data (ortho: orthopohoto of Debrecen; WV2: WorldView-2 image of Debrecen) (blue dotted line: 95% benchmark)

Regarding the OPPs, the orthopohoto of Debrecen did not provide 100% purity (i.e. when all pixels of a given roof were correctly classified); usually there were no roofs in this category, the only exception being the red tile, although its OPP was below 1% (Fig. 11a–d). In comparison, the WV2 image provided better results, with OPPs ranging from 30 to 55% for red tiles and 10–24% for dark tiles and asbestos, depending on the validation technique. At the 75% purity class, the average OPPs were above 60% for the orthophotos and 84% for the WV2 image across all validation techniques. In the 51% purity class, OPPs had an average of 94% for WV2 and >86% in the case of the orthophoto. Regarding the four purity classes, the satellite image outperformed the orthophotos; however, the differences decreased relevantly in lower purity classes (32–36% at 90% purity and 5–8% at 51% purity). Dark tiles generally performed worse than asbestos and red tile.

Considering the rankings of the 144 models (4 purity levels × 3 roofing types × 6 classification approaches × 2 types of input data), we revealed that the first 5 had 100% OPP, 51% purity and IT and TT validation techniques, and the first two belonged to the asbestos class and the other three were red tiles. 29 models met the 95% OPP benchmark (Fig. 11a–d), 13 were asbestos, 16 were red tiles, while the dark tiles did not feature in the list (appearing later in the ranking only in 36th place); 2 of the models were of 90% (Fig. 11c), 6 models of 75% (Figs. 11b) and 21 models of 51% pixel purity (Fig. 11a). Among these models the validation techniques had the following distribution: 7 cases of IT and TT, 5 cases of SCV2, 4 cases of CVCent and CVSeg, and 2 cases of SCV10.

The GLM revealed a non-significant model (Table 3) where the adjusted R^2^ was only 1.4%. Accordingly, regarding the object-based post-classification, validation type, the interaction between validation type and roofing type had no significant influence on OPPs, but the role of the roofing type, with a small effect, was significant.

**Table 3:** Result of GLM performed on OPP values as the dependent variable and the validation type and roofing type as independent variables (SS: sum of squares, df: degree of freedom, F: F-statistic, p: significance, ω^2^p: effect size).

Parameters	SS	df	F	p	ω^2^p
Model	2.401	17	1.122	0.340	0.014
Validation type	0.323	5	0.514	0.766	−0.017
Roof type	1.824	2	7.246	0.001	0.080
Validation type × roof type	0.254	10	0.202	0.996	−0.059
Residuals	15.855	126			
Total	65.249	144			

## 4 Discussion

Accuracy assessment is an essential part of all classifications. Although RKCV is an effective tool in evaluating model performance, due to the large number of resulting models, it can be reliable only in datasets without autocorrelation [52]. Although in remote sensing spatial autocorrelation is a common feature of the images and the assumption of data independence can be violated, it can be handled in several ways (e.g. random point sampling, design-based sampling, or using segments). The issue of independence arises especially when reference data collection is based on cluster sampling; thus, the results are misleading AA-metrics, which overestimate the model's performance [18,[53], [54], [55]]. Our target objects - roofs - represented a special case in which the possible areas to collect reference data were limited to houses; thus, the possible method to avoid, i.e. using segments, was not a perfect solution. Roofs have a small extent; thus, segments are also small with possible autocorrelation, and can facilitate the overestimation of the OAs with all types of input data. This was the case with our data, too: CVSeg was better than TT or CV, but MIs indicated high SA (Fig. 7). Due to SA, OAs were 7–17% higher compared to the independent measurements. We justified that applying a completely (spatially) independent dataset is an appropriate way to avoid the negative effects of SA and our results were similar to the findings of Bahn and McGill [53], Ibrahim and Bennett [19] and Ploton et al. [56]. Setting the threshold sampling distance parameter to a sufficiently large value (in our case 10 m) removes the effects of positive SA; thus, RKCV is not biased and can be a reliable validation technique of thematic accuracy. It also justified our hypothesis that spatial-based pixel sampling helps to avoid the influence of SA. However, this type of spatial sampling relevantly reduces the number of reference data, which increases the uncertainty of the model, i.e., widens the confidence intervals (Fig. 7; [57]).

When all reference data were used (TT), the minimum difference between the predicted and actual accuracy was achieved with the WV2 image (7.5%) while in the case of the orthophoto of Vaihingen it was 15%. The explanation lies in the difference in spatial resolution - the WorldView-2 image (50 cm after pan-sharpening) was considerably lower than the ortophotos (9 cm): the difference between data volumes was nearly 31-fold, thus, roofs were represented in more pixels. The accuracies of the different samplings (Fig. 7) were consistent with the findings of Underwood et al. [58]: better results can be achieved with higher spectral over spatial resolution in certain cases due to the increase in intra-class variation of pixels with finer spatial resolution. This corresponds with the results of Marceau et al. [59], Tran et al. [60], and Sun et al. [61], i.e. the negative effects of higher intra-class variation can be reduced by using images with coarser spatial resolution, leading to higher classification accuracies especially in urban areas with high heterogeneity. Visually, outcomes of pixel-based classifications usually suffered from the salt-and-pepper effect, i.e. the interspersing of misclassified pixels among the correct ones, but there were also buildings of 100% purity.

Calculations on OPP quantified the level of the salt-and-pepper effect: a purity ratio of 100% meant that the classified image was homogeneous, while a purity threshold of 51% indicated that the level of homogeneity on a given roof did not exceed 49%. Roofs are built from the same material, but there are several inhomogeneities such as roof windows, antennae, shadows, lichens and mosses, etc.; therefore, the occurrence of interspersed other classes of pixels is normal; however, the majority of the pixels can define the real class. 100% purity is rather a theoretical target value, only a few buildings reached this condition, but the 51% purity class was an appropriate choice, having the highest OPPs in most models. The main finding is that although OAs were relevantly overestimated due to SA, the class level metric (OPP) showed different results; however, while OAs were calculated based on pixels, OPPs were based on the objects after a post-classification and this procedure eliminated the detrimental influence of SA. Differences among the OPPs were not significant according to the 2-way ANOVA (Table 2). As OPP decreases with the spatial resolution, the salt-and-pepper effect becomes more prominent in the classified image causing lower accuracies and noisier thematic maps.

The best results were obtained with the WV2 satellite image, which has a coarser spatial resolution (50 cm) than the orthophotos (<10 cm); accordingly, relative standard deviations of the orthophotos were ∼5% larger than the WV2 image (13.3 [WV2] vs. 19.0 [ortho Debrecen] and 18.3 [ortho Vaihingen]). Among the roofing types, dark tiles had a lower performance, which was caused by the highest pixel variation (especially in the case of the Debrecen orthophoto), as this category incorporates tiles of various shades of black, brown and grey. The lower accuracies of the Vaihingen orthophoto can be attributed to the fact that the quality of the image is generally worse (higher noise levels and distortions, data gaps, etc), and roofs are more heterogeneous because of the large number of solar panels.

A great concern is that when reducing the number of training pixels, the accuracy decreases, too, and most importantly, the OPP also decreased. Thus, the two main findings are (i) although SA influences the accuracy measures, the final outcome on the object level is still accurate; (ii) although SCV2 and SCV10 did not ensure the most accurate map, the number of training pixels was very low (38–83). In spite of the very low number of training pixels, SCV10 was efficient: both the influence of SA was minimized and an acceptable accuracy was gained, and after the majority-pixel-based post-classification the class level accuracies hardly differed from the most accurate IT model, at least with the WV-2.

One limitation of our approach to spatial sampling is that it requires a certain amount of data, which is sometimes not feasible. Particularly for smaller objects (e.g. roofs), this type of spatial sampling can significantly reduce the data volume, thus increasing the uncertainty of the models (i.e. resulting in wider confidence intervals); thus, a situation may occur in which, although the model is not affected by SA, it does not provide the best classification map.

## 5 Conclusions

The aim of this study was to quantify the overestimation of the classification accuracies due to high positive SA and also to propose a method that can eliminate its negative effects, thus, making RKCV a reliable validation technique. Our results revealed that.

• SA's effect was significant on the classification accuracy of a random split of reference data into training and testing subsets and with RKCV, as it was reflected in the differences between the difference related to the independent reference data: high positive MI can bias the accuracies by up to 17%, which falsely resulted in OAs above 99%, and the result is a high proportion of misclassified pixels.
• Using image segments to reduce the number of pixels was not sufficient to eliminate SA due to the limited extent of the roofs - selected pixels were still autocorrelated.
• The proposed method, the resampling of reference data based on their spatial location, successfully eliminated the bias in the accuracy when estimating the model's performance with RKCV; accuracies were in the range of independent testing.
• The WV2 image with coarser spatial resolution (50 cm) outperformed the orthophotos (9 and 10 cm) by 5–9% due to the lower intra-class variance. Furthermore, the higher relative standard deviation also contributed to the increased noise in the thematic maps caused by the salt-and-pepper effect.
• Using the building polygons in a majority-pixel-based object-oriented post-classification we found that all sampling techniques ensured >95% OPPs.

Collecting training and testing data in image classification is an eligible method, but in this case the output will be only a single model with occasionally changing accuracy. RKCV performs well in statistical modelling, but remotely sensed data is highly biased by SA. Our method is a possible solution to overcome the SA-issue, which allows the use of RKCV in special cases of classification in which target objects have a limited extent and the object-oriented methods do not perform well.

## Author contribution statement

David Abriha; Szilard Szabo: Conceived and designed the experiments; Performed the experiments; Analyzed and interpreted the data; Contributed reagents, materials, analysis tools or data; Wrote the paper.

Prashant Srivastava: Analyzed and interpreted the data; Wrote the paper.

## Funding statement

This work was supported by the Doctoral Student Scholarship Program of the Co-operative Doctoral Program of the Ministry of Innovation and Technology; NKFI K 138079, K 142121, and TKP2020-NKA-04.

## Data availability statement

Data will be made available on request.

## Declaration of interest's statement

The authors declare no conflict of interest.

## Appendix A Supplementary data

deb_orto_purity deb_orto_purity

vaihingen_orto_purity vaihingen_orto_purity

wv2_purity wv2_purity
